# High prevalence of undisclosed antiretroviral drug use among individuals initiating HIV treatment in Gaborone, Botswana

**DOI:** 10.3389/fpubh.2025.1582940

**Published:** 2025-06-05

**Authors:** Natasha O. Moraka, Sikhulile Moyo, Terence Mohammed, Kesaobaka Molebatsi, Lubbe Wiesner, Patrick T. Mokgethi, Irene Gobe, Margaret Mokomane, Salang T. Moutswi, Laone Rabatoko, Queen Leteemane, Vanessa Strachan-Amaro, Phenyo Sabone, Simani Gaseitsiwe

**Affiliations:** ^1^Botswana Harvard Health Partnership, Gaborone, Botswana; ^2^Faculty of Health Sciences, School of Allied Health Professions, University of Botswana, Gaborone, Botswana; ^3^Department of Immunology and Infectious Diseases, Harvard University T.H Chan School of Public Health, Boston, MA, United States; ^4^Faculty of Sciences, School of Health Systems and Public Health, University of Pretoria, Pretoria, South Africa; ^5^Division of Medical Virology, Department of Pathology, Faculty of Medicine and Health Sciences, Stellenbosch University, Cape Town, South Africa; ^6^Division of Clinical Pharmacology, Department of Medicine, University of Cape Town, Cape Town, South Africa

**Keywords:** antiretroviral drugs (ARVs), antiretroviral therapy (ART), human immunodeficiency virus (HIV), Botswana, newly HIV diagnosed individuals, undisclosed ARV drug use

## Abstract

**Background:**

Antiretroviral therapy (ART) uptake is critical for evaluating the effectiveness of HIV epidemic control. We evaluated the extent of undisclosed ARV drug use among individuals newly diagnosed and initiating ART in greater Gaborone, Botswana.

**Methodology:**

Plasma samples from an ongoing longitudinal cohort study were screened for antiretroviral drug (ARV) traces using the liquid chromatography with tandem mass spectrometry assay. The ARV drug screening panel used detects 4 ARV drugs: Integrase Strand Transfer Inhibitor [INSTI]—dolutegravir (DTG), two non-nucleoside reverse transcriptase inhibitors [NNRTIs]—Efavirenz (EFV), Nevirapine (NVP), and a protease inhibitor [PI]—Lopinavir. We estimated adjusted prevalence ratios (aPR) for factors associated with undisclosed ART use using modified Poisson regression.

**Results:**

We enrolled 192 participants, between October 2023 and January 2024, and a total of 120 (63.4%) were screened for plasma ARV drug traces. Participants were of median age 32 (IQR 26, 39), mostly female (66.7%) and of Botswana nationality (75.0%). Among those screened for ARV drug traces 36 (30.0%; 95%CI: 30–39) participants had at least one of the ARVs in the panel detected. One participant (0.8%) was positive for EFV, and 35 (29%) had DTG traces at baseline. Undisclosed ART use was associated with lower viral load (aPR = 0.84; 95%CI: 0.70–1.00) and being of non-Motswana nationality (aPR = 2.6; 95%CI: 1.5–4.5).

**Conclusion:**

We report a relatively high proportion of individuals with undisclosed drug use in their baseline plasma. Our results suggest the need to implement pre-drug screening for routine HIV incidence surveillance, including pre-treatment drug resistance evaluations before ART initiation.

## Introduction

Accurate estimates of the human immunodeficiency virus (HIV) diagnoses and antiretroviral therapy (ART) use among people living with HIV (PLWH) are essential for monitoring progress toward the UNAIDS fast-track targets for controlling the HIV pandemic by 2030 (1). Botswana is among the first African countries to introduce free ART to all HIV-diagnosed individuals regardless of clinical markers or residency status as per the “Treat All” strategy (2).

There are several presumptions regarding the “Treat all” strategy, including that every newly HIV-diagnosed individual has no prior exposure to ART (3), and their transmitted founder virus strain is likely susceptible to the first-line treatment in their country/region. However, the issue of undisclosed ART use among newly HIV-diagnosed individuals may severely impact treatment outcomes usually associated with defaulting and re-initiation; thus impacting programmatic incidence estimates, pre-treatment drug resistance, and further HIV treatment and care (4–7). In Botswana, it was previously reported that 39% of individuals who reported no history of ART and low (<400 copies/mL) or undetectable HIV-1 viral load (VL) at baseline had traces of ARVs in their plasma (8).

Consequently, it is important to determine the proportion of individuals who have a history of ART exposure prior to ART initiation for accurate national and regional ART uptake estimates. We sought to evaluate the extent of undisclosed ART use among newly HIV-diagnosed individuals government clinics in Greater Gaborone, Botswana.

## Methodology

### Study population and ethical considerations

Whole blood samples were obtained from participants in the “Tekodiso study,” an ongoing longitudinal cohort study tracking individuals recently diagnosed with HIV at government clinics in the Greater Gaborone area of Botswana (October 2023–November 2024). The study employed a consecutive census approach, enrolling all individuals diagnosed with HIV through double rapid testing on the same day of their diagnosis, before the initiation of ART. Ethical approval was received from the University of Botswana Institutional Review Board (REF NO. UBR/RES/IRB/BIO/357), the Botswana Ministry of Health; Health Research and Development Division (HRDC, REF NO. HPRD: 6/14/1), as well as the Greater Gaborone DHMT [REF NO. GGDHMT 6/17/1 IV (61)].

### HIV-1 viral load testing

HIV-1 VL in plasma was quantified using Abbott m2000sp/Abbott m2000rt (Abbott Laboratories, Wiesbaden, Germany) following the manufacturer’s instructions. The lower limit of detection for this assay is 40 copies/mL.

### Antiretroviral drug (ARV) screening

The ARV drug screening panel used detects 4 ARV drugs; Integrase Strand Transfer Inhibitor [INSTI]—dolutegravir (DTG), two non-nucleoside reverse transcriptase inhibitors [NNRTIs]—Efavirenz (EFV) and Nevirapine (NVP), as well as the protease inhibitor [PI]—Lopinavir (LPV). A high-performance liquid chromatography with tandem mass spectrometry assay was developed and validated for screening dolutegravir, efavirenz, lopinavir, and nevirapine in human plasma. The assay was developed at the Division of Clinical Pharmacology, University of Cape Town. Sample preparation consisted of a protein precipitation extraction procedure followed by high-performance liquid chromatography with tandem mass spectrometry detection using gradient elution. Dolutegravir-d4, efavirenz-d5, lopinavir-d8, and nevirapine-d3 were used as internal standards. A Sciex API 4000 mass spectrometer at unit resolution in the multiple reaction monitoring mode was used to monitor the transition of the protonated precursor ions at m/z 420.1, 316.0, 629.5, and 267.1 to the product ions m/z 277.2, 243.9, 447.2, and 226.0 for dolutegravir, efavirenz, lopinavir, and nevirapine, respectively. The QC cut-off concentration for the positivity of each analyte was set at 0.02 μg/mL to allow for adequate ARV detection. Two sets of quality control samples were analysed during inter- and intra-validation (2 × n = 18). The percentage difference for all drugs was less than 2.6% between sets, and the coefficient of variation was less than 11.5%.

### Statistical analysis

Proportions were estimated with 95% confidence intervals (CI) using the binomial exact method. Adjusted prevalence ratios (aPR) and 95% CI were estimated for factors associated with undisclosed ART using modified Poisson regression with robust standard errors. Variables with *p*-values less than 0.2 in univariate models were included in the multivariate regression analysis to adjust for possible confounding, including age and gender *a priori*. *p*-values less than 0.05 indicated significance. We compared the characteristics of those with ARV analyte data and those without using Fishers exact tests for categorical variables and Student t-test or Wilcoxon signed-rank test for quantitative variables. Statistical analyses were performed in STATA v.18 (College Station, TX).

## Results

A total of 192 participants were enrolled between October 2023 and January 2024. Participants self-reported no prior ARV use on the day of diagnosis. At the time of the analysis, a total of 120 participants were screened for the presence of ARVs. A total of 36 (30.0, 95%CI: 21.9–39.0%) were positive for any ARVs, and 35 (29.2, 95%CI: 21.2–38.2%) were positive for DTG. Among these, 25 (71.4, 95%CI: 53.7–85.4%) had detectable HIV RNA at baseline, and 10 (28.6, 95%CI: 14.3–46.3%) had undetectable HIV RNA (< 40 copies/mL), Table 1. More females had detectable DTG traces (27/80, 34%) than males (8/40, 20%). Nineteen (52.7%) participants with DTG detected were non-citizens, with the rest being Batswana nationals (Table 1). One (0.8%) male participant, with a suppressed viral load and a CD4 count of 548 cells/mL tested positive for EFV. None of the participants were positive for NVP or LPV. Undisclosed ART use was only associated with being of non-Motswana nationality (aPR = 2.59; 95% CI: 1.48–4.54) as well as lower HIV RNA load (aPR = 0.84; 95%CI: 0.70–1.00) (Table 1). When comparing those with DTG analyte data and those without, we did not observe any statistically significant differences in gender (*p* = 0.28), nationality (*p* = 0.19), marital status (*p* = 0.48) or mean age (*p* = 0.28).

**Table 1:** Predictors of undisclosed drug use among newly HIV-1 diagnosed individuals in Gaborone, Botswana.

Characteristic (*N* = 120)	Proportion, *n* (%)	ARV drug detected (*n* = 36)	PR (95% CI)	*p*-value	aPR (95% CI)	*p*-value
Sex, *n* (%)						
Male	40 (33.3)	8 (20.0)	0.7 (0.3–1.3)	0.1	0.6 (0.3–1.1)	0.08
Female	80 (66.7)	27 (33.8)	1 (ref)			
Age in years (Q1, Q3)	32 (26, 39)	33 (27, 41)	1.0 (0.99–1.0)	0.3	1.0 (0.98–1.0)	0.3
Education, *n* (%)						
None/primary/ non formal	8 (6.7)	4 (11.1)	0.6 (0.3–1.2)	0.2		
Secondary/tertiary	112 (93.3)	30 (83.3)				
Citizenship, *n* (%)						
Non-Motswana	30 (25.0)	17 (47.2)	3.2 (1.9–5.4)	<0.01	3.0 (1.5–4.5)	<0.01
Employment, *n* (%)						
Employed	80 (66.7)	24 (66.7)	0.1 (0.5–1.7)	0.9		
HIV viral load (log10 copies/mL)	4.3 (3.6, 4.9)	4.1 (1.6, 4.6)	0.7 (0.6–0.9)	<0.01	0.84 (0.70–1.00)	0.03
CD4 (cells/mL)	362 (218, 524)	324 (194, 567)	1.0 (1.0–1.0)	0.8		

ARV, antiretroviral drugs; aPR, adjusted prevalence ratio; CD4, cluster of differentiation 4; HIV, human immunodeficiency virus; PR, prevalence ratio; RNA, ribonucleic acid.

## Discussion

We report high levels of undisclosed ART use among individuals initiating ART in Greater Gaborone, Botswana, who self-reported no prior exposure to ARVs at the time of first HIV diagnosis. This is comparable with a previous report of ART naïve virally suppressed individuals in Botswana during the country’s transition to “Treat all” (8). These results potentially have implications for estimates for ART uptake and monitoring of the treatment targets, such as the UNAIDS 95-95-95, tracking of new infections or transmitted drug resistance. Drug resistance surveillance, where unrecognised ART exposure may affect baseline resistance patterns if these individuals are assumed to be treatment-naïve.

At baseline, we report the highest detection of DTG (29% DTG and 0.8% EFV) and no detection of NVP and LPV, suggesting no exposure to previous or current second-line ARV regimen. We observed close to a third of individuals with undetectable HIV RNA (<40 copies/mL) having a detection of DTG. Previous studies have reported that having undetectable plasma HIV-1 RNA at baseline while self-reporting no pre-ART exposure is a potential marker of undisclosed ART use (9). Furthermore, high levels of DTG traces could be an indication of individuals being exposed to first-line regimens and defaulting, leading to re-initiation into the national ARV program. Although this data is not shown, none of our participants admitted to taking any pre-exposure prophylaxis at the time of initiation. Several factors have been attributed to defaulting, however, it is difficult to determine the true reason for defaulting in public health settings, particularly with a one-pill first-line regimen which is relatively easier to adhere to than earlier regimens.

Some of the factors previously associated with undisclosed ARV drug use include stigma, defaulting/non-adherence, denial of first HIV test results or shared drug use which contains ARVs (8). Studies within the region have reported that younger people are at higher risk of HIV acquisition and non-adherent behaviour, leading to undisclosed ART use (4, 6, 8). However, this was not the case in our study, probably due to a higher age distribution in our study population. Undisclosed drug use was associated with non-national citizenship in our study, which is in agreement with previous reports indicating that migration patterns lead to poor healthcare access, which may lead to defaulting and re-initiation among non-citizens (10). Low and suppressed HIV VL has also been reported to be a common factor among individuals with undisclosed ART use (5).

Our study had a few limitations; firstly, we did not account for individual drug metabolism rates when detecting each drug, which could confound the results observed. A positive drug trace may only reflect the drug used within the past few weeks, and hence some participants with no drug trace may have taken ARVs and long-interrupted treatment. This discordance between biomarker detection and self-report is a key phenomenon we aim to highlight, as it has important implications for HIV incidence and transmitted drug resistance estimation. Our sampling was also limited to a few high-volume facilities in the capital city. However, our sampling area targeted the highest ART enrolment facilities within the region and the methodology used to determine drug traces had quality checks optimal enough to allow for adequate ARV detection and accurate individual drug-based results.

## Conclusion

We report high levels of undisclosed ARV use among newly HIV-diagnosed individuals within the greater Gaborone area, Botswana. Undisclosed ARV drug use remains a major public health concern even when interventions and free ART are offered within public healthcare facilities, further impeding the success of ART uptake estimates and reducing accurate denominators to track the UNAIDS 95-95-95 target success. We recommend deliberate efforts to obtain ART history as well as drug tracing especially in surveys for HIV incidence and pre-treatment drug resistance.

## Data Availability

The raw data supporting the conclusions of this article will be made available by the authors, without undue reservation.
